# Complete genome sequence of *Capnocytophaga* strain ARDL2 from human blood infection

**DOI:** 10.1128/mra.00912-24

**Published:** 2024-12-17

**Authors:** Alec Victorsen, Aidan Ellison, Jeffrey Kubiak, Patricia Ferrieri, Bharat Thyagarajan, Evann E. Hilt

**Affiliations:** 1 Department of Laboratory Medicine and Pathology, University of Minnesota, Minneapolis, Minnesota, USA; Wellesley College Department of Biological Sciences, Wellesley, Massachusetts, USA

**Keywords:** *Capnocytophaga*

## Abstract

This paper describes the genome assembly of a *Capnocytophaga* strain ARDL2 isolated from a human blood infection. The genome assembly comprised one circular chromosome with the length of 2,439,195 bp with a G+C content of 34.81%.

## ANNOUNCEMENT

*Capnocytophaga* species are commensal organisms in the oral cavity of both humans and animals (1, 2). *Capnocytophaga* species from canines and felines can be transmitted to humans through a bite, scratch, or salivary contact with pre-existing wounds, yet the clinical presentation can vary depending on the natural host for particular species, making species-level identification critical (2, 3). While the most common zoonotic *Capnocytophaga* species are *C. canimorsus*, *C. canis*, and *C. cynodegmi*, other *Capnocytophaga* species have been isolated, although their pathogenicity remains unknown (4, 5).

This announcement reports the complete genome of a *Capnocytophaga* species isolated from a human bloodstream infection. Blood culture bottles were collected from an immunocompromised patient whose pet dog licked a leg wound. The blood culture bottles were flagged positive on the BACT/Alert system (BD, Sparks, MD), and organisms from the bottles had a Gram stain suspicious for *Capnocytophaga*. The initial subculture from the aerobic bottle required 7 days of growth on buffered charcoal yeast extract (BCYE) agar (Remel, Alexa, KS) at 37°C, where the organism grew as pinpoint colonies. The organism was not tested biochemically, failed to thrive for antimicrobial susceptibility testing, and could not be identified with MALDI-TOF mass spectrometry. Sanger sequencing was performed on ~500 bp of the 16S bacterial ribosomal RNA from isolated colonies, yielding a product most closely resembling *Capnocytophaga* sp. feline oral taxon 300 strain 7171 (accession KM461964.1, percent identity 97.25%).

Bacteria cultured on BCYE agar (as described above) were suspended in 400 µL water and homogenized on a FastPrep-24 Classic using 2.0 mL Lysing Matrix B tubes (MP Biomedicals, Santa Ana, CA) twice for 30 seconds at 6.0 m/s, interrupted with a 5-minute rest on ice before centrifugation at 2,000 *g* for 5 minutes. DNA was isolated from the supernatant using the Maxwell Viral Total Nucleic Acid Purification Kit (Promega, Madison, WI) using the manufacturer’s instructions. The isolated DNA was eluted in 60 µL of water and not sheared or size-selected. Libraries were prepped using the Nanopore Ligation Sequencing kit v14 [Oxford Nanopore Tech. (ONT), Oxford, UK] with 32 µL DNA and sequenced on a Nanopore PromethION-24 with a 10.4.1 flow cell for 16 hours. For bioinformatics analysis, default parameters were used except where otherwise noted. Raw POD5 files were converted into uBAM format using Dorado (v0.7.1, model: dna_r10.4.1_e8.2_400bps_sup@v5.0.0). All default options in Dorado were used to perform the QC, error calling, adapter trimming, and base calling. N50 was calculated using NanoStat (v1.5.0). Contigs were generated using the ONT’s wf-bacterial-genome workflow (v0.3.0) (6, 7).

Sequencing results and the annotation are summarized in Table 1. The four 16S sequences are identical to JN713507.1 (*Capnocytophaga* sp. Canine oral taxon 339 clone 1I154 16S ribosomal RNA gene, partial sequence). There was no substantial homology between the assembled genome and any published *Capnocytophaga* genomes. The assembled genome has a total length of 2,439,195 bp, with an overall G + C content of 34.81%. BUSCO (v5.4.3) run in the genome mode against the bacterial lineage, odb10, gave a completeness score of 98.4% (8). The annotation reported 2,404 coding sequences from 2,491 genes. The assembled genome was flagged as circular by Flye and confirmed by querying the fastq for the relevant sequence.

**TABLE 1:** Summary of sequence data and genome features

Metric	Value
Oxford Nanopore	
Total number of reads sequenced	5.36 M
N50 (kb)	4.8
Average contig read depth	8,642 x
Assembled genome^*a*^	
Number of contigs	1
Total genome size (no. of bases)	2,439,195
G + C content (%)	34.81
Genome completeness by BUSCO (%)	98.4
Number of genes	2,491
Number of CDSs	2,404
Number of tRNAs	73
Number of rRNAs	13

^*a*^ Circular chromosome.

We present a *Capnocytophaga* species likely originating from canine oral flora. This genome submission increases the awareness of non-*canimorsus Capnocytophaga* species with pathogenic potential.

## Data Availability

The Nanopore sequence reads and genome sequence have been deposited in SRA and GenBank databases under the accession number: CP168034.1 (BioProject: PRJNA1140139, BioSample: SAMN42812522) and SRA number: SRX25701811.
